# Diverse clinical presentations of neurosyphilis: focus on differential diagnosis

**DOI:** 10.1007/s10072-017-3204-2

**Published:** 2017-11-27

**Authors:** Agnieszka Chrząszcz-Włodarczyk, Adam Włodarczyk, Joanna Szarmach

**Affiliations:** 1 Department of Opthalmology, Medical University of Gdansk, Dębinki 7 St. build. 25, 80–952 Gdansk, Poland; 2 Department of Psychiatry, Medical University of Gdansk, Dębinki 7 St. build. 25, 80–952 Gdansk, Poland

To the Editor,

We read with interest the paper by Serrano-Cardenas et al. addressing the issue of uncommon clinical manifestation of neurosyphilis presenting with cognitive decline and mood disorders as a primary symptoms. The article discusses a rare presentation of neurosyphilis emphasizing the importance of atypical clinical symptomatology of the disorder [1]. However, the discussion of that interesting paper left almost unaddressed an issue of neuropsychiatric evaluation in differential diagnosis in neurosyphilis.

Włodarczyk et al. recently reported on two cases of neurocognitive decline as a primary symptom of neurosyphilis including a patient with a year-long history of visual acuity worsening in right eye optic nerve neuropathy with hypertensive angiopathy of second degree (in both eyes) being associated with neurosyphilis [2]. The sight loss preceded the neurocognitive decline being hypothetically indicative of neurosyphilis onset manifesting with the worsening of sight due to optic nerve neuropathy that is the symptom hardly associated with neurosyphilis and may be missed in differential diagnosis. In line with Serrano-Cardenas et al.s’ findings [1], the brain MRI demonstrated abnormal findings with particular focus on the apparent diffusion coefficient (ADC) measure value being pronounced in the patient with optic nerve neuropathy [3].

The variable course and pleomorphic forms of neurosyphilis are challenging medical issue as atypical forms may mimic the proper diagnosis. It is particularly true for psychiatric presentations of neurosyphilis disclosing at first depression, cognitive impairment, psychomotor retardation, suicidal ideation, or decreased appetite, sleep, and energy [4].

As neurosyphilis is a diagnostic challenge the conclusive paper by Dr. Serrano-Cardenas and co-workers (2017) is of vital interest reviewing the syphilitic encephalitis along with neuropsychiatric aspects of the disease. However, along with the described course, clinical symptoms, brain MRI findings the ophthalmologic examination may provide earlier premises for widening the patients testing when standard examination does not fully address the burden of the disease [5]. As the diagnosis of neurosyphilis is complex the importance of multidisciplinary screening of high-risk groups such as patients with neuropsychiatric symptomatology is of prime importance. In our opinion, brain MRI and ophthalmic examination may well contribute to the adequate management of neurosyphilis in the process of the differential diagnosis.
